# ECAE: An Efficient Certificateless Aggregate Signature Scheme Based on Elliptic Curves for NDN-IoT Environments

**DOI:** 10.3390/e27050471

**Published:** 2025-04-26

**Authors:** Cong Wang, Haoyu Wu, Yulong Gan, Rui Zhang, Maode Ma

**Affiliations:** 1 College of Artificial Intelligence, Tianjin University of Science and Technology, Tianjin 300457, China; wangcongjcdd@tust.edu.cn (C.W.); 1981215263@mail.tust.edu.cn (H.W.); gyllaile@mail.tust.edu.cn (Y.G.); zhangrui1113@mail.tust.edu.cn (R.Z.); 2 College of Engineering, Qatar University, Doha P.O. Box 2713, Qatar

**Keywords:** named data networking, internet of things, elliptic curve cryptography, certificateless aggregate signature, message authentication

## Abstract

As a data-centric next-generation network architecture, Named Data Networking (NDN) exhibits inherent compatibility with the distributed nature of the Internet of Things (IoT) through its name-based routing mechanism. However, existing signature schemes for NDN-IoT face dual challenges: resource-constrained IoT terminals struggle with certificate management and computationally intensive bilinear pairings under traditional Public Key Infrastructure (PKI), while NDN routers require low-latency batch verification for high-speed data forwarding. To address these issues, this study proposes ECAE, an efficient certificateless aggregate signature scheme based on elliptic curve cryptography (ECC). ECAE introduces a partial private key distribution mechanism in key generation, enabling the authentication of identity by a Key Generation Center (KGC) for terminal devices. It leverages ECC and universal hash functions to construct an aggregate verification model that eliminates bilinear pairing operations and reduces communication overhead. Security analysis formally proves that ECAE resists forgery, replay, and man-in-the-middle attacks under the random oracle model. Experimental results demonstrate substantial efficiency gains: total computation overhead is reduced by up to 46.18%, and communication overhead is reduced by 55.56% compared to state-of-the-art schemes. This lightweight yet robust framework offers a trusted and scalable verification solution for NDN-IoT environments.

## 1. Introduction

The widespread adoption of Internet of Things (IoT) smart devices across various domains has profoundly transformed daily life [1,2,3]. However, the massive interconnection of devices and the exponential growth of data volumes have intensified critical challenges, such as data security threats and transmission efficiency bottlenecks, imposing new demands on communication security and performance [4]. To address these issues, Named Data Networking (NDN) has emerged as a promising content-centric communication paradigm due to its inherent advantages [5]. Unlike traditional address-based network architectures, NDN focuses on the data itself through a name-based routing mechanism, offering enhanced flexibility, scalability, and built-in security. Nevertheless, deploying NDN in IoT environments introduces complex security challenges arising from heterogeneous device capabilities, including identity authentication, message integrity assurance, and privacy protection [6,7,8].

In the NDN architecture, digital signatures serve as a fundamental mechanism for data packet verification. By embedding cryptographically generated unique identifiers into data packets, digital signatures ensure content authenticity and integrity, playing a pivotal role in preventing data source spoofing, tampering, and man-in-the-middle attacks [9]. However, this mechanism must satisfy the following two critical requirements: (1) IoT terminal devices, constrained in computational power and storage, struggle to handle the certificate chain validation and bilinear pairing operations required by traditional Public Key Infrastructure (PKI); (2) NDN routers must support low-latency and batch signature verification in high-speed data forwarding scenarios.

Traditional PKI relies on digital certificates to bind user identities to public keys. However, the communication overhead associated with certificate issuance, storage, and revocation imposes significant burdens on resource-constrained IoT terminals. Furthermore, the risks of single points of failure in certificate authorities (CAs) and the complexity of cross-domain authentication limit PKI’s applicability in distributed IoT environments. Certificateless Cryptography (CLC) provides a compelling alternative by integrating Identity-Based Cryptography (IBC) with partial private key distribution through a Key Generation Center (KGC). This approach eliminates the need for certificate management while avoiding the key escrow problem inherent in IBC. In this study, we propose a key generation mechanism that incorporates partial private key distribution, ensuring data packet security while reducing computational costs.

Moreover, the data-centric nature of NDN may result in concurrent responses from multiple producer nodes for a single request. When using conventional signature schemes, routers must verify each data packet independently, leading to computational overhead that scales linearly with the number of packets. Aggregate signature (AS) techniques address this limitation by compressing independent signatures into a fixed-length aggregate result, thereby reducing verification complexity to a constant level and significantly enhancing router processing efficiency. This study further innovates by combining elliptic curve cryptography (ECC) with universal hash functions to eliminate the need for bilinear pairings, achieving substantial improvements in verification performance.

### 1.1. Related Work

In recent years, research on security in Named Data Networking-Based Internet of Things (NDN-IoT) has primarily focused on achieving two key objectives: protecting data integrity and ensuring data source authentication. Existing solutions can be broadly classified into four main categories: (1) signature authentication schemes based on edge computing [10,11]; (2) blockchain-based signature authentication schemes [12,13,14,15]; (3) certificateless signature authentication schemes [16,17,18,19]; (4) batch signature authentication schemes [20,21,22,23].

By offloading computational tasks to edge nodes, these approaches reduce the core network load and enhance overall system performance. For instance, Wang et al. [10] proposed the NIoTE framework, which improves data retrieval efficiency from neighboring service nodes through requested aggregation and distributed caching. Huang et al. [11] introduced a certificateless group signature scheme; however, its reliance on computationally intensive bilinear pairing operations resulted in high computational costs, limiting its practicality in large-scale deployments. Moreover, the scheme’s centralized edge gateway architecture for identity management presents a security vulnerability—if the gateway is compromised, regional data verification could be undermined.

In blockchain-based authentication schemes, Lou et al. [12] proposed a distributed key management mechanism for NDN, leveraging blockchain to store hashed public keys in the NDN Testbed model, thus reducing the certificate chain verification time during user authentication. However, since blockchain stores data in transaction records, retrieving specific transactions requires traversing the entire block, which may lead to increased average retrieval delays as the number of transactions grows. Lei et al. [13] integrated cache poisoning protection and privacy-aware access control into a blockchain-based NDN network, but the approach’s computational overhead restricts its application to vehicular networks. Alsamhi et al. [14] combined federated learning with blockchain to protect model data and verify human–machine interactions, ensuring privacy and security. More recently, Yang et al. [15] proposed a blockchain-based encryption scheme that authenticates IoT devices and encrypts signals. However, its dependence on bilinear pairing operations leads to a significantly higher computational overhead than ECC-based alternatives.

Certificateless signature schemes eliminate certificate management, provide lightweight solutions for resource-constrained environments, and significantly reduce management costs. Karati et al. [16] proposed a lightweight pairing-based signature scheme for IoT, but its security still relies on computationally expensive operations. Rezaeibagha et al. [17] enhanced Karati’s model from a security perspective but failed to resolve the efficiency bottleneck caused by pairing. Thumbur et al. [18] utilized the Elliptic Curve Discrete Logarithm Problem (ECDLP) for security; however, their complex ECC-based algorithm remained unsuitable for constrained environments. Bisheh-Niasar et al. [19] presented an efficient FPGA-based implementation of Ed25519, utilizing Karatsuba multiplication and parallelism to accelerate signing. While this provides hardware-level optimization, it lacks sufficient algorithm-level resistance against attacks.

To alleviate the burden on resource-limited IoT devices, researchers have also explored batch signature authentication. Asami et al. [20] proposed an NDN aggregation signature scheme based on proof-of-concept identity verification to reduce communication overhead. This scheme enhances the efficiency of both signature generation and verification using aggregate signatures. Nonetheless, identity- and attribute-based schemes that rely on key escrows continue to pose critical security risks. Zhang et al. [21] introduced a multi-party authentication scheme based on a centralized certificate authority, drawing criticism due to its centralized trust model. Xue et al. [22] proposed a cooperative authentication framework using a name-prefix-based public key binding for distributed router verification, yet the model still depends on a vulnerable central authority. Zhu et al. [23] developed a reconfigurable certificateless signature scheme to prevent dishonest data modification and improve bandwidth efficiency. However, its trade-off between security and efficiency remains an issue.

Although existing research has advanced NDN-IoT authentication technologies across various dimensions, several core challenges remain—particularly in balancing security with efficiency, improving attack resilience, and building a secure and trustworthy communication environment. Current solutions face three major limitations: (1) performance degradation due to bilinear pairing or other complex cryptographic computations when scaling to large numbers of connected devices; (2) dependence on centralized certificate authorities in batch signature schemes, which introduces single points of failure; and (3) insufficient anonymity, exposing users to traceability and privacy risks.

These challenges underscore the urgent need for novel authentication mechanisms tailored to resource-constrained NDN-IoT environments—mechanisms that can simultaneously address security, efficiency, and privacy concerns. Developing a highly secure, lightweight communication framework capable of resisting diverse attacks is crucial for enabling practical deployment in such environments.

### 1.2. Motivation

To ensure the core security properties of data integrity, authentication, and access control are upheld and to establish a more secure and trustworthy communication environment, researchers have proposed a variety of communication security schemes aimed at defending against data tampering and unauthorized access. Despite these efforts, existing approaches still present significant limitations. For instance, in edge computing-based signature authentication schemes, such as the work by Huang et al. [11], edge computing enhances local performance but fails to guarantee the legitimacy of producer data packets in cases where the edge gateway is compromised. In blockchain-based signature authentication schemes, Lou et al. [12], Lei et al. [13], and Yang et al. [15] improved communication security through the use of blockchain. However, due to blockchain’s inherent efficiency bottlenecks and the computational overhead of bilinear pairing operations, these solutions remain unsuitable for large-scale deployment scenarios. In the domain of certificateless signature schemes, Karati et al.’s [16] scheme has been shown to be vulnerable to signature forgery attacks during security analysis. While Thumbur et al. [18] strengthened data security by leveraging the Elliptic Curve Discrete Logarithm Problem (ECDLP), the scheme’s computational complexity leads to reduced efficiency and increased communication overheads, particularly in resource-constrained environments. Regarding batch signature verification schemes, Xue et al. [22] proposed a cooperative content authentication framework that enables verification across multiple routers. However, their approach heavily depends on third-party trust centers, thereby introducing potential security threats to end consumers and IoT devices. Experimental data show that, under typical conditions, a single bilinear pairing operation on an elliptic curve incurs approximately 20 times the computational cost of a point multiplication operation. Moreover, hash function mappings to elliptic curve points are significantly more time-consuming than standard cryptographic hash functions. As the number of terminal devices increases, the size of aggregated signatures grows linearly, severely impacting communication efficiency and introducing considerable storage overheads. To address these challenges, this paper proposes a pairing-free and efficient signature authentication scheme, which demonstrates both strong security guarantees and high computational efficiency, making it well-suited for deployment in resource-constrained NDN-IoT environments.

### 1.3. Contribution

The main contributions of this paper are as follows:

- This paper proposes an efficient certificateless aggregate signature scheme based on elliptic curve cryptography (ECC) in the NDN-IoT environment, named ECAE. To accommodate resource-constrained devices, ECAE eliminates the need for computationally expensive map-to-point hash functions and bilinear pairing operations, thereby improving system computational efficiency. Furthermore, by implementing aggregate signatures, ECAE ensures that the signature length remains constant regardless of the number of terminal devices, effectively reducing communication overhead.
- ECAE adopts a key generation mechanism that integrates identity authentication with partial private key distribution, satisfying security functional requirements and ensuring secure communication.
- ECAE demonstrates strong security against various attacks in both theoretical analysis and simulations while also exhibiting high computational efficiency and low communication overheads.

## 2. Preliminaries

### 2.1. System Architecture

**Figure 1:**
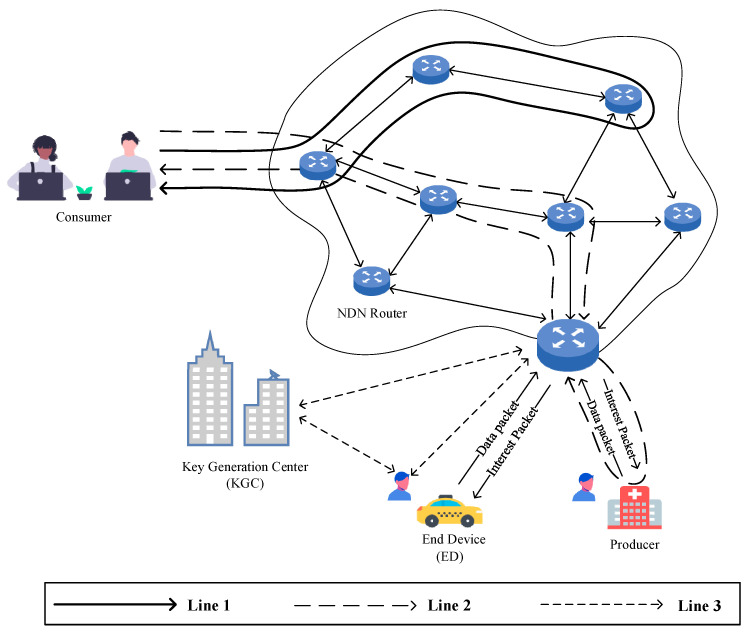
System network model diagram.

This paper proposes an efficient and conditionally confidential certificateless aggregate signature scheme for NDN-IoT, establishing a network model that consists of four main entities: the Key Generation Center (KGC) and NDN routers, consumers, and producers. The network model is illustrated in Figure 1.

- Line 1 represents an example of the process in which a consumer requests data forwarding from an NDN router.
- Line 2 illustrates the process of a consumer requesting data packets from multiple producers.
- Line 3 demonstrates the interaction between terminal devices (producers in Figure 1) and the KGC for registration and the process of generating an aggregate signature and transmitting data packets via the NDN router.

When an Edge Device (ED) requests to join the network as a producer or consumer in the NDN-IoT system, it first transmits its real identity to the Key Generation Center (KGC) through a secure channel for registration. Subsequently, the KGC assigns a partial private key and public parameters to the ED. Each ED possesses a unique real identity along with a corresponding public–private key pair and must obtain a signature from the KGC before transmitting any message. Upon receiving a message–signature pair, the NDN router verifies the signature to ensure its validity and maintain message integrity. The roles of the key entities in the system are described as follows:

- KGC (Key Generation Center): The KGC acts as the public parameter distribution authority and key management center for the entire system. During the system’s initialization phase, it is responsible for generating and publishing system parameters. When a new entity requests to join the system, it must securely interact with the KGC to obtain public parameters, pseudonyms, and partial private keys.
- NDN Router: The NDN router verifies the authenticity and integrity of data packets during transmission. As a critical component ensuring secure data forwarding, it performs signature verification on the encapsulated producer information within the data packet. Additionally, it aggregates digital signatures from multiple terminal devices to enhance computational efficiency.
- Consumer: The consumer serves as the data requester in the proposed system, corresponding to the consumer entity in NDN. It requests the required data or services by sending Interest packets.
- Producer: The Producer is responsible for data generation in the NDN-IoT environment, corresponding to the producer entity in NDN. It utilizes sensor devices to collect information such as soil moisture, vehicle location, and indoor temperature.

### 2.2. Elliptic Curve Cryptography

Elliptic curve cryptography (ECC) is a public-key cryptographic algorithm based on the mathematical principles of elliptic curves. Compared to traditional cryptographic algorithms (e.g., RSA), ECC offers higher performance and smaller key sizes for the same level of security, making it particularly suitable for resource-constrained environments such as mobile and IoT devices.

The fundamental principle of ECC involves the addition and multiplication of points on an elliptic curve, enabling key generation, data encryption and decryption, and cryptographic operations such as digital signatures. Its security is based on the Elliptic Curve Discrete Logarithm Problem (ECDLP). Given a prime-order finite field $\;Z_{q}^{*}$, an elliptic curve G is defined as follows: $G=\left\{\left(x,y\right)|y^{2}=x^{3}+ax+b,a,b\in Z_{q}^{*}\right\}\cup \left\{O\right\}$, where $4a^{3}+27b^{2}mod\;p\neq 0$, O represents the point at infinity, and p is a prime number. The addition and multiplication of points on an elliptic curve are defined as follows:

- For any point, $P(x_{1},y_{1})\in G$; then, $P(x_{1},y_{1})+O=O+P(x_{1},y_{1})=P(x_{1},y_{1})$.
- For two points, $P\left(x_{1},y_{1}\right),Q(x_{2},y_{2})\times G$; if $x_{1}=x_{2}$ and $y_{1}=-y_{2}$, then $P\left(x_{1},y_{1}\right)+Q\left(x_{2},y_{2}\right)=0$.
- If $P\left(x_{1},y_{1}\right),P(x_{1},y_{1})$ and P=±Q, then $P\left(x_{1},y_{1}\right)+Q\left(x_{2},y_{2}\right)=R(x_{3},y_{3})$, where $x_{3}=\lambda_{2}-x_{1}-{\;x}_{2}$ and $y_{3}=\lambda \left(x_{1}-x_{3}\right)-y_{1}$, The definition of λ can be found in Equation (1).
  (1) $$=\left\{\begin{array}{c} \frac{y_{2}-y_{1}}{x_{2}-x_{1}}\left(mod\;p\right),if\;P\neq Q\\ \frac{3x_{1}^{2}+a}{2y_{1}}\left(mod\;p\right),if\;P=Q \end{array}\right.$$
- Scalar multiplication is defined as follows: $lP=P_{1}+\ldots +P_{l}$, where $l\in Z_{q}^{*}$ and l>0. ECDLP: Given two points, P,Q∈G, it is computationally unfeasible to find an integer $c\in Z_{q}^{*}\;$ such that Q=cP holds.

### 2.3. Scheme Framework

The commonly used identifiers in the implementation of this scheme are shown in Table 1.

The implementation process of the proposed scheme mainly includes the following six steps:

- KGC System Initialization: The security parameters are input λ. The KGC initializes the system, generates the master key s  and system params, and then keeps the master key,  s,  confidential while publishing the system params.
- Interaction between KGC and ${ED}_{i}$ to Generate Public and Private Keys:$\;{ED}_{i}$ sends its real identity ${ID}_{i}$ to the KGC. The KGC runs an algorithm offline to generate the pseudonym ${PID}_{i}$; then, ${ED}_{i}$ selects a random value $x_{i}\in Z_{q}^{*}$ as its key value and sends the pseudonym ${PID}_{i}\;$ to the KGC. The KGC runs an algorithm to generate the partial private key for ${ED}_{i}$. Using the key value and the private key sent by the KGC as inputs, ${ED}_{i}$ generates its secret key ${SK}_{i}$ and public key ${PK}_{i}$.
- The terminal device ${ED}_{i}$ signature generation: $\;{ED}_{i}$ uses ${PID}_{i}$, $m_{i}\in {\left\{0,1\right\}}^{*}$ and ${SK}_{i}$ as inputs to generate a signature $\sigma_{i}.$
- Single signature verification: the ${ED}_{i}$ or NDN router uses ${PID}_{i}$, $m_{i}\in {\left\{0,1\right\}}^{*}$, $\sigma_{i}$ and ${PK}_{i}$ as the inputs. If $\sigma_{i}$ is valid, the output is accepted; otherwise, the output is rejected.
- The NDN router performs signature aggregation: The NDN router compresses the different signatures $\sigma_{i}(i=1,\;2,\ldots ,n)$ from n data packets requested by the same consumer from n different producers within the same time period into a single signature σ.
- Other NDN routers perform aggregate signature verification: They take $\left\{{PID}_{i},m_{i},\sigma_{i},{PK}_{i}\right\}(i=1,\;2,\ldots ,n)$ as the input. If σ is valid, the output is accepted; otherwise, the output is rejection.

**Figure 2:**
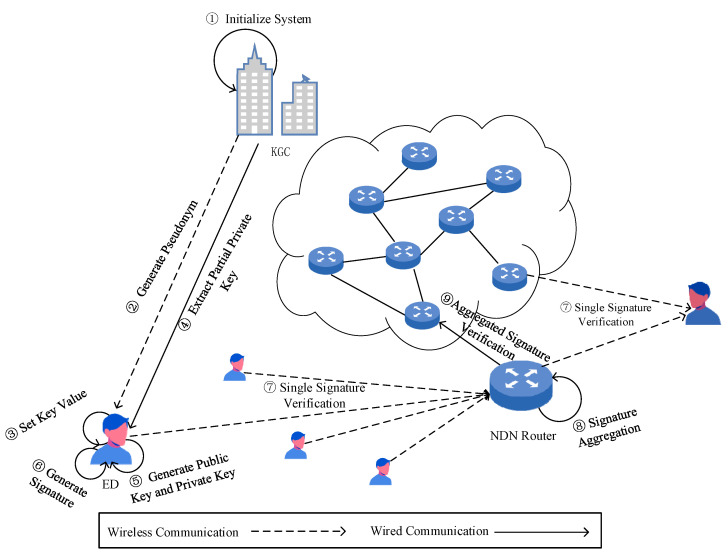
System process framework of the scheme.

The system process framework of this scheme is shown in Figure 2.

### 2.4. Security Model

In the proposed scheme, we consider Type I and Type II attackers as defined in [24]. Due to the lack of certificate verification, an attacker may replace an entity’s public key with a public key of their choice. The two types of attackers are simulated as follows:

- Type I attacker: This attacker simulates an external adversary who can replace any entity’s public key with a specific value chosen by the attacker. However, a Type I attacker does not know the private key of the Key Generation Center (KGC).
- Type II attacker: This attacker simulates a malicious KGC that has access to the master key but cannot replace the public keys of other entities.

Furthermore, both Type I and Type II attackers can be classified into three attack levels [25,26]:

- An ordinary adversary can only learn valid verification messages.
- A strong adversary can replace public keys to forge valid verification messages, provided that the attacker possesses the corresponding private value.
- A super adversary can obtain valid verification messages for a public key that is replaced without submitting any content.

In general, a super adversary may issue the following queries:

- create-user (${PID}_{t}$): This oracle model takes ${PID}_{t}$ as the input, where ${PID}_{t}\;$ represents the identity of the t-th terminal device. It then runs the relevant algorithm to obtain the partial private key, $D_{t}$, the key value,$x_{t}$, and the public key, ${PK}_{t}$.
- request-public-key (${PID}_{t}$): This oracle model takes ${PID}_{t}$ as the input. It searches the list L and returns the public key ${PK}_{t}$ of the t-th terminal device.
- replace-public-key (${PID}_{t},{PK}_{t},{PK}_{t}^{\prime }$): This oracle model takes (${PID}_{t},{PK}_{t},{PK}_{t}^{\prime }$) as the input. It replaces the public key of the t-th terminal device with ${PK}_{t}^{\prime }$ and updates the corresponding information in list L.
- extract-secret (${PID}_{t}$): This oracle model takes ${PID}_{t}$ as the input. It searches the list L and returns the key value $x_{t}$. However, if the t-th terminal device has been queried with a replace-public-key request, it returns null.
- extract-partial-secret (${PID}_{t}$): This oracle model takes ${PID}_{t}$ as the input. It then searches the list, L, and returns the partial private key, $D_{t}$.
- super-sign (${PID}_{t},m_{t}$): This oracle model takes (${PID}_{t},m_{t}$) as the input, where $m_{t}$ represents the message to be signed. The oracle outputs a signature $\sigma_{t}=(R_{t},U_{t},V_{t})$, such that $true\leftarrow Verify(m_{t,}\sigma_{t},params,{PID}_{t},{PK}_{t}).$ If the public key has not been replaced, i.e., ${PK}_{t}={PK}_{t}^{\prime }$, ${PK}_{t}$ is the public key returned by the oracle model request-public-key (${PID}_{t}$). Otherwise, ${PK}_{t}={PK}_{t}^{\prime }$, where ${PK}_{t}^{\prime }$ is the latest public key value submitted to the oracle model replace-public-key (${PID}_{t},{PK}_{t},{PK}_{t}^{\prime }$).

The following two games, namely Game 1 and Game 2, are designed for Super Type I and Super Type II adversaries, respectively. Super Type I adversary simulates an external adversary who can replace any entity’s public key with a specific value of their choice. Super Type II adversary simulates a malicious KGC that holds the master key and may engage in adversarial activities such as eavesdropping on signatures and issuing signature queries.

Game 1. This game is played between the challenger, C, and the Super Type I adversary ${SA}_{1}$, where they interact within the proposed certificateless signature scheme. First, in the “Initialization” phase, challenger C runs the corresponding algorithm and generates the private key s, along with the public system parameters params. Then, C keeps s, secret while providing params to adversary ${SA}_{1}.$ Second, in the “Query” phase, SA1 can adaptively access the following oracle queries: create-user (${PID}_{t}$), request-public-key (${PID}_{t},{PK}_{t},{PK}_{t}^{\prime }$), replace-public-key (${PID}_{t}$), extract-secret (${PID}_{t}$), extract-partial-secret (${PID}_{t}$), and super-sign (${PID}_{t},m_{t}$). After making all necessary queries, ${SA}_{1}\;$ outputs a forged signature (${PID}_{t},m_{t},\sigma_{t}$). ${SA}_{1}$ wins Game 1 if the following three conditions hold:

- ${SA}_{1}$ has never queried the oracle extract-partial-secret (${PID}_{t}$).
- ${SA}_{1}$ has never queried the oracle super-sign (${PID}_{t},m_{t}$).
- $true\leftarrow Verify(m_{t,}\sigma_{t},params,{PID}_{t},{PK}_{t})$, where ${PK}_{t}$ is the current public key of the t-th terminal device, which can be replaced by ${SA}_{1}$.

**Definition** **1.** *The proposed certificateless signature scheme is existentially unforgeable against a Super Type-I adversary ${\;SA}_{1}$ if, within polynomial time, ${SA}_{1}\;$ undertakes the following: $q_{H}$ queries to the hash oracle, ${\;q}_{CU}\;$ queries to create-user (${PID}_{t}$), ${\;q}_{EPS}$ queries to extract-partial-secret (${PID}_{t}$), $q_{ES}$ queries to extract-secret (${PID}_{t}$), $q_{PK}$ queries to request-public-key (${PID}_{t}$), ${\;q}_{RPK}$ queries to replace-public-key (${PID}_{t},{PK}_{t},{PK}_{t}^{\prime }$), and ${\;q}_{SS}$ queries to super-sign (${PID}_{t},m_{t}$* *). If ${Succ}_{SA1}$, the probability of $\;{SA}_{1}$ winning Game 1, is negligible, and the scheme is secure against a Super Type-I adversary.*

Game 2. This game takes place between a challenger C and a Super Type-II adversary ${SA}_{2}$, interacting within the proposed certificateless signature scheme. First, in the “Initialization” phase, challenger C runs the corresponding algorithms and generates a private key s, along with public system parameters params. Then, C keeps s secret while providing params to the adversary. Second, in the “Query” phase, ${SA}_{2}$ can adaptively access the following oracle queries: create-user (${PID}_{t}$), request-public-key (${PID}_{t},{PK}_{t},{PK}_{t}^{\prime }$), replace-public-key (${PID}_{t}$), extract-secret (${PID}_{t}$), extract-partial-secret (${PID}_{t}$), and super-sign (${PID}_{t},m_{t}$). After making all necessary queries, ${SA}_{2\;}$ outputs a forged signature (${PID}_{t},m_{t},\sigma_{t}$). ${SA}_{2\;}$ wins Game 2 if the following three conditions hold:

- ${SA}_{2}$ has never queried the oracle extract-secret (${PID}_{t}$).
- ${SA}_{2}$ has never queried the oracle super-sign (${PID}_{t},m_{t}$).
- $true\leftarrow Verify\;(m_{t,}\sigma_{t},params,{PID}_{t},{PK}_{t})$, where ${PK}_{t}$ is the current public key of the t-th terminal device, which can be replaced by ${SA}_{1}$.

**Definition** **2.** *The proposed certificateless signature scheme is existentially unforgeable against a Super Type-II adversary ${\;SA}_{2}$ if, for any polynomial-time adversary ${\;SA}_{2}$, the success probability ${Succ}_{SA2\;}$ of winning Game 2 is negligible. ${SA}_{2}$ is allowed to undertake the following:${\;q}_{H}$ queries to the hash oracle, $q_{CU}$ queries to create-user (${PID}_{t}$), $q_{EPS}$ queries to extract-partial-secret (${PID}_{t}$), $q_{ES}$ queries to extract-secret (${PID}_{t}$), $q_{PK}$ queries to request-public-key (${PID}_{t}$), $q_{RPK}$ queries to replace-public-key (${PID}_{t},{PK}_{t},{PK}_{t}^{\prime }$), and $q_{SS}$ queries to super-sign (${PID}_{t},m_{t}$). If ${Succ}_{SA2}$ is negligible, then the scheme is secure against a Super Type-II adversary.*

### 2.5. Security Requirements

In the NDN-IoT environment, an efficient certificateless aggregate signature scheme based on elliptic curves should meet the following security requirements:

- Data Integrity: Elliptic curve cryptography (ECC) should be used to protect data from unauthorized access during transmission. Encryption ensures that only legitimate entities possessing the corresponding decryption key can access and interpret the data, effectively maintaining the privacy and confidentiality of communications.
- Key Management: The secure generation, distribution, and updating of keys is essential to prevent key leakage or misuse. A robust key management system is crucial for maintaining overall system security and ensuring the safety and validity of cryptographic keys.
- User Privacy: In an NDN-IoT system, content names are used to request data, and these names are semantically related to user preferences, making them visible within the network. Additionally, since each node in an NDN-IoT system can cache content, malicious users may still obtain content even if content names are protected. Therefore, user privacy protection must be ensured by maintaining content anonymity and protecting user behavior privacy, preventing attackers from inferring the content of user requests.
- Resistance to Various Security Attacks: The NDN-IoT system is vulnerable to multiple security threats, such as impersonation and replay attacks. Thus, the proposed signature scheme must be capable of resisting various security attacks to ensure the safety and reliability of communications.

## 3. Proposed Scheme

This paper proposes an efficient certificateless aggregate signature scheme based on ECC, utilizing ECC and a general hash function while avoiding bilinear pairing operations and map-to-point hash functions.

Meanwhile, considering a resource-constrained environment, we introduce an unauthenticated aggregate signature method. The proposed scheme enables cryptographic signature aggregation for n concurrent message transmissions. A consumer sends requests to n producers over a period of time, with each producer generating a data packet. The original independent signatures from the n data packets are compressed into an aggregate signature within the NDN router, thereby reducing signature verification time and minimizing the storage overhead of the NDN router.

### 3.1. System Initialization

The KGC takes a security parameter, $\lambda \in Z^{*}$, as the input and selects a cyclic group G of prime order q, where P is a generator of G. Next, the KGC randomly selects a secret value $s\;\in \;Z_{q}^{*}$ and computes the public key:${\;P}_{pub}=sP$. Then, the KGC sets s as the master secret key and $P_{pub}$ as the public key. The KGC also selects three general one-way hash functions: $H1:\;{\left\{0,1\right\}}^{*}\times G\to Z_{q}^{*}$, $H2:\;{\left\{0,1\right\}}^{*}\times G\times G\to Z_{q}^{*}$, $H2:\;{\left\{0,1\right\}}^{*}\times G\times G\to Z_{q}^{*}$, $H2:\;{\left\{0,1\right\}}^{*}\times G\times G\to Z_{q}^{*}$. Finally, the KGC keeps s confidential and publishes the system parameters: $params=\{q,\;G,\;P,\;P_{pub},\;H1,\;H2,\;H3\}$.

### 3.2. Generation of Terminal Device Pseudonyms

**Figure 3:**
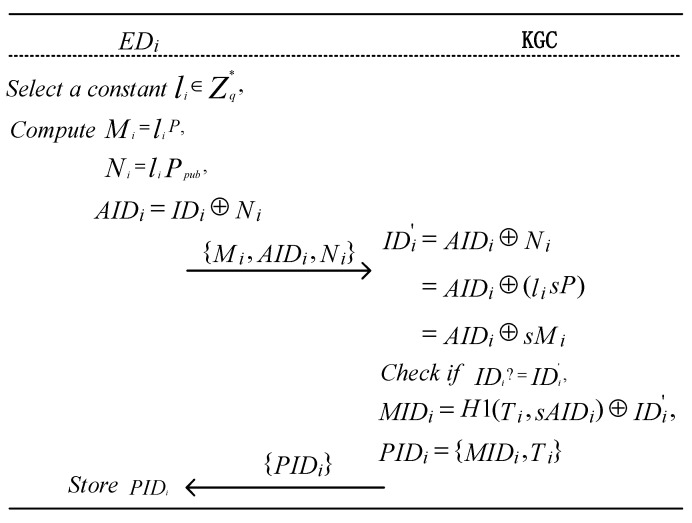
Process of generating a pseudonym for terminal devices.

To prevent malicious users from launching traffic analysis attacks or user location tracking attacks in the NDN network, each terminal device ${ED}_{i}$ must register its real identity ${ID}_{i}$ with the KGC to generate a pseudonym for anonymous communication. Additionally, since the KGC manages the real identities, ${ID}_{i}$, of all terminal devices, ${ED}_{i}$, in the system, it can effectively track malicious users. The terminal device ${ED}_{i}$ randomly selects a value $l_{i}\in Z_{q}^{*}$ and computes the following: $M_{i}={\;l}_{i}P,\;N_{i}={\;l}_{i}P_{pub},\;{AID}_{i}={ID}_{i}⊕N_{i}$ Then, ${ED}_{i}$ submits $\left\{M_{i},\;{AID}_{i},N_{i}\right\}$ to the KGC. Upon receiving $\left\{M_{i},\;{AID}_{i},N_{i}\right\}$, the KGC computes the following: ${ID}_{i}^{\prime }={AID}_{i}⊕N_{i}={AID}_{i}⊕\left(l_{i}sP\right)={\;AID}_{i}⊕sM_{i}$ Thus, the real identity of the terminal device is retrieved. If ${ID}_{i}^{\prime }$ is invalid, the KGC discards the request. Otherwise, the KGC computes the following: ${MID}_{i}=H1(T_{i},s{AID}_{i})\;⊕{ID}_{i}^{\prime }$ and then sends ${PID}_{i}=\{{MID}_{i},T_{i}\}$ to ${ED}_{i}$, where $T_{i}$ represents the validity period of the pseudonym ${PID}_{i}$. The implementation process is illustrated in Algorithm 1 and Figure 3.


**Algorithm 1: Generation of *ED_i_* Pseudonym**
Input: *params*, *ID_i_*Output: *PID_i_*1.        $l_{i}\in Z_{q}^{*}$        //Generate a random constant2.        *M_i_ = l_i_ P*;3.        *N_i_ = l_i_ P*_pub_;4.        ${AID}_{i}={ID}_{i}⊕N_{i}$;5.        $s\;\in \;Z_{q}^{*}$        //Generate a random constant6.        ${ID}_{i}^{\prime }={AID}_{i}⊕N_{i}={AID}_{i}⊕\left(l_{i}sP\right)={AID}_{i}⊕sM_{i}$;7.        $\mathrm{if}\;({ID}_{i}^{\prime }\;=={\;ID}_{i}$) then8.                ${MID}_{i}=H1(T_{i},s{AID}_{i})\;⊕{ID}_{i}^{\prime }$; T_i_ is the validity period of the PID_i_9.                ${PID}_{i}=\{{MID}_{i},T_{i}\}$;10.       else return null;11.       $\mathrm{return}\;{ED}_{i}\leftarrow {PID}_{i}$

To track the real identity of terminal devices, the KGC stores $\left\{{PID}_{i},M_{i},{AID}_{i}\right\}$ in its secure database. In the future, these terminal devices will communicate using pseudonyms, which can help protect their real identity information.

### 3.3. Generation of Terminal Device Keys

When ${ED}_{i}$ obtains its pseudonym from the KGC, it selects a random number $x_{i}\in Z_{q}^{*}$ as its key value and computes $X_{i}=x_{i}P$. Then, ${ED}_{i}$ sends its pseudonym ${PID}_{i}$ to KGC. The KGC searches for ${PID}_{i}$ in its database. If it exists, the KGC executes the following algorithm to generate the partial private key for ${ED}_{i}$.

Given the public parameters, params, and the terminal device pseudonym, ${PID}_{i}$, the KGC selects a random number $r_{i}\in Z_{q}^{*}$ and computes $R_{i}=r_{i}P$. The KGC then computes the following: $h_{2i}=H2\left({PID}_{i},R_{i},P_{pub}\right),d_{i}=r_{i}+sh_{2i}(mod\;q)$ The partial private key $D_{i}=(d_{i},R_{i})$ is then sent to ${ED}_{i}$. Upon receiving $D_{i}$, the terminal device ${ED}_{i}$ verifies whether the following equation holds: $d_{i}P=R_{i}+h_{2i}P_{pub}.$ If the equation holds, ${ED}_{i}$ accepts the partial private key. Otherwise, it rejects it.

**Figure 4:**
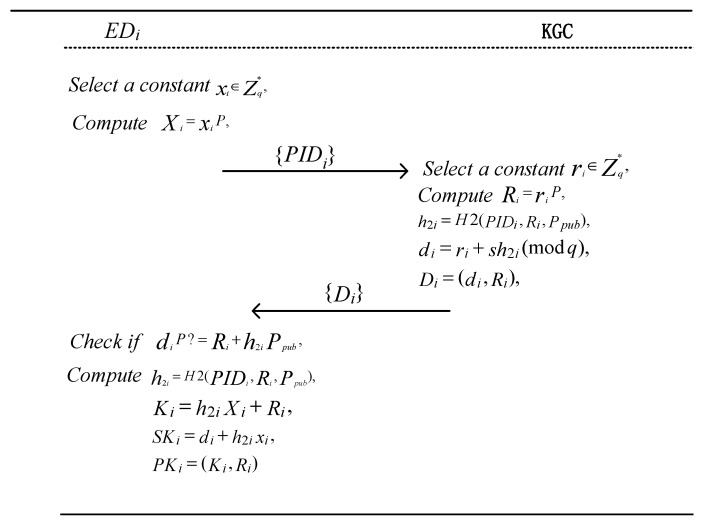
Process of generating terminal device keys.

Finally, ${ED}_{i}$ computes $h_{2i}=H2\left({PID}_{i},R_{i},P_{pub}\right),K_{i}=h_{2i}X_{i}+R_{i}$ Then, it sets ${SK}_{i}=d_{i}+h_{2i}x_{i}$ as its private key and ${PK}_{i}=\left(K_{i},R_{i}\right)\;$ as its public key. The implementation process is illustrated in Algorithm 2 and Figure 4.


**Algorithm 2: Generation of *ED_i_* Key**
Input: *params*, *PID_i_*Output: *SK_i_, PK_i_*1.        $x_{i}\in Z_{q}^{*}$;        //Generate a random constant2.        $X_{i}=x_{i}P$;3.        $r_{i}\in Z_{q}^{*}$;        //Generate a random constant4.        $R_{i}=r_{i}P$;5.        $h_{2i}=H2({PID}_{i},\;R_{i},P_{pub})$;6.        $d_{i}=r_{i}+sh_{2i}(mod\;q)$;7.        if ($d_{i}P\;==R_{i}+h_{2i}P_{pub}$) then8.              $h_{2i}=H2({PID}_{i},R_{i},P_{pub})$;9.              $K_{i}=h_{2i}X_{i}+R_{i}$;10.            ${SK}_{i}={\;d}_{i}+h_{2i}x_{i}$;11.            ${PK}_{i}=(K_{i},R_{i})$;12.      else return null;13.      $\mathrm{return}\;\{{SK}_{i},{PK}_{i}\}$

### 3.4. Generation and Verification of a Single Digital Signature

To generate a digital signature, ${ED}_{i}$ obtains a message: $m_{i}\in {\left(0,1\right)}^{*}$. It then selects a random number, $u_{i}\in Z_{q}^{*}$, and computes the following: $U_{i}=u_{i}P,h_{3i}=H3\left(m_{i},{PID}_{i},{PK}_{i},U_{i},t_{i}\right)$ where $t_{i}$ is the current timestamp. ${ED}_{i}$ then computes $V_{i}=u_{i}+h_{3i}{SK}_{i}$. Thus, the output signature is $\sigma_{i}=(U_{i},V_{i})$. Finally, ${ED}_{i}$ sends the information $\left\{{PID}_{i},m_{i},{PK}_{i},\sigma_{i},t_{i}\right\}$ to a nearby NDN router. The process is described in Algorithm 3.


**Algorithm 3: Generation of a Single Digital Signature**
Input: *params*, *PID_i_*, *SK_i_*, *PK_i_*Output: *σ_i_*1.        $m_{i}\in {\left(0,1\right)}^{*}$;2.        $u_{i}\in Z_{q}^{*}$;3.        $U_{i}=u_{i}P$;4.        $h_{3i}=H3\left(m_{i},{PID}_{i},{PK}_{i},\;U_{i},\;t_{i}\right)$;      //*t_i_* is the current timestamp5.        $V_{i}=u_{i}+h_{3i}{SK}_{i}$;6.        $\sigma_{i}=(U_{i},V_{i})$;7.        $\mathrm{return}\;\mathrm{NDN}\;\mathrm{Router}\;\leftarrow \;\sigma_{i}$;

When performing digital signature verification, or when the NDN router receives the message $\left\{{PID}_{i},m_{i},{PK}_{i},\sigma_{i},t_{i}\right\}$, it first checks whether $t_{i}$ is within the valid period, $T_{i}$, and whether $t_{i}$ is fresh. If it is invalid, the message is discarded; otherwise, the NDN router computes $h_{2i}=H2\left({PID}_{i},R_{i},P_{pub}\right),h_{3i}=H3\left(m_{i},{PID}_{i},{PK}_{i},t_{i}\right)$ and verifies whether the equation $V_{i}P=U_{i}+h_{3i}(K_{i}+h_{2i}P_{pub})$ holds. If the equation holds, the NDN router accepts the signature, and the single digital signature verification is successful; otherwise, the signature is rejected.

### 3.5. Generation and Verification of Aggregated Digital Signatures

When an NDN router receives a large number of messages $\left\{{PID}_{i},m_{i},{PK}_{i},\sigma_{i},t_{i}\right\}$ from different ${ED}_{i}(i=1,2,\ldots ,n)$, it computes $V=\sum_{i=1}^{n}V_{i}$ and $U=\sum_{i=1}^{n}U_{i}$; it then outputs the aggregated signature σ=(U,V). Finally, the NDN router sends $\left\{m_{i},{PID}_{i},{PK}_{i},t_{i}\}(i=1,\;2,\ldots ,n\right)$ along with σ to other NDN routers. The algorithm process is shown in Algorithm 4.


**Algorithm 4: Generation of Aggregated Digital Signature**
Input: *σ_i_*Output: *ς*1.        $V=\sum_{i=1}^{n}V_{i}$;2.        $U=\sum_{i=1}^{n}U_{i}$;3.        σ=(U, V);4.        return NDN Routers ←σ;

When other NDN routers receive $\left\{m_{i},{PID}_{i},{PK}_{i},t_{i}\}(i=1,\;2,\ldots ,n\right)$ and σ, the NDN routers check the validity of the aggregated signature σ by verifying whether $VP=U+\sum_{i=1}^{n}h_{3i}(K_{i}+h_{2i}P_{pub})$ holds, where $h_{2i}=H2({PID}_{i},R_{i},P_{pub})$ and $h_{3i}=H3\left(m_{i},{PID}_{i},{PK}_{i},t_{i}\right)$. If the equation holds, the aggregated signature is accepted, and the verification is successful; otherwise, the signature is rejected, and the verification fails.

## 4. Security Analysis

### Correctness Analysis

The proof of the correctness of single signature verification is shown in Equation (2):

(2) $$\begin{array}{cc} V_{i}P & =\left(u_{i}+h_{3i}{SK}_{i}\right)P\\ & =u_{i}P+h_{3i}\left(d_{i}+{\;h}_{2i}x_{i}\right)P\\ & ={\;U}_{i}+h_{3i}\left(d_{i}P+h_{2i}x_{i}P\right)\\ & ={\;U}_{i}+h_{3i}\left(R_{i}+h_{2i}P_{pub}+h_{2i}X_{i}\right)\\ & =U_{i}+h_{3i}(K_{i}+h_{2i}P_{pub}) \end{array}$$

The proof of correctness of the aggregate signature verification is shown in Equation (3):

(3) $$\begin{array}{cc} VP & =\sum_{i=1}^{n}V_{i}P\\ & =\sum_{i=1}^{n}\left(U_{i}+h_{3i}\left(K_{i}+h_{2i}P_{pub}\right)\right)\\ & =\sum_{i=1}^{\;n}U_{i}+\sum_{i=1}^{n}h_{3i}\left(K_{i}+{\;h}_{2i}P_{pub}\right)\\ & =U+\sum_{i=1}^{n}h_{3i}(K_{i}+h_{2i}P_{pub}) \end{array}$$

## 5. Formal Security Analysis

Under the condition that solving the ECDLP is extremely difficult, we have proven that the ECAE scheme possesses existential unforgeability against both Super Type I and Super Type II adversaries.

**Theorem** **1.** *In the random oracle model, assuming that solving the ECDLP is difficult, the proposed certificateless signature scheme possesses existential unforgeability against a Super Type-I adversary. That is, if there exists a Super Type-I adversary* *${SA}_{1}$ that can submit queries to the random oracle model and win Game 1 with probability ${Succ}_{{SA}_{1}}$, then there exists an algorithm β  that can solve a random instance of the ECDLP in polynomial time, with a success probability of ${Succ}_{\beta }\;\geq \;\frac{1}{q_{CU}+q_{H}}{\left(1-\frac{1}{q_{CU}+{\;q}_{H}}\right)}^{q_{EPS}}{Succ}_{{SA}_{1}}$*

**Proof.**  Assume there exists a Super Type-I adversary ${SA}_{1}$ that can break our proposed certificateless signature scheme with a non-negligible probability ${Succ}_{{SA}_{1}}$. We can construct a polynomial-time algorithm β that utilizes ${SA}_{1}$ to solve the ECDLP.

Initialization Phase: Algorithm β initializes two tables: a hash table, $L_{H1}$ (initially empty), and a key table, $L_{K1}$ (initially empty). In Game 1, β selects an identity ${PID}^{*}$ as the challenge identity, sets the public key of the KGC as ${PK}_{KGC}$, and sends the parameters $params=(G,P,{PK}_{KGC},H)$ to ${SA}_{1}$.

Query Phase of the Super Adversary:

- Create-user (${PID}_{t}$): The random oracle model takes ${PID}_{t}$ as the input. If ${PID}_{t}$ has already been created, nothing happens. Otherwise, β runs the corresponding algorithm to generate the following: the partial private key $D_{t}$, the secret key $x_{t}$, and the public key ${PK}_{t}$. Then, β returns these values to ${SA}_{1}$.
- Hash Queries: (1) When ${SA}_{1}$ queries the hash function at $\left({PID}_{t},{PK}_{KGC}\right)$, if the list $L_{H1}$ contains $<h_{t},{PID}_{t},{PK}_{KGC}>$, then β returns $h_{t}$ to ${SA}_{1}$. Otherwise, β randomly selects $h_{t}\in Z_{n}^{*}$, returns it to ${SA}_{1}$, and adds $<h_{t},{PID}_{t},{PK}_{KGC}>$ to $L_{H1}$. (2) When ${SA}_{1}$ queries the hash function at $\left(m,{h_{t},PID}_{t},T_{t}\right)$, if $L_{H1}$ contains $<k_{t},m,{h_{t},PID}_{t},T_{t}>$, then β returns $k_{t}$ to ${SA}_{1}$. Otherwise, β randomly selects $k_{t}\in Z_{n}^{*}$, returns it to ${SA}_{1}$, and adds $<k_{t},m,{h_{t},PID}_{t},T_{t}>$ to $L_{H1}$.
- request-public-key (${PID}_{t}$): Upon receiving a request-public-key query for identity ${PID}_{t}$, β checks if ${PID}_{t}\neq {PID}^{*}$. If true, β selects three random values $a_{t},b_{t},x_{t}\in Z_{n}^{*}$ and computes the following: $s_{t}\leftarrow a_{t},h_{t}\leftarrow b_{t},R_{t}\leftarrow a_{t}P-b_{t}{PK}_{KGC},{PK}_{t}=x_{t}P+R_{t}$. β updates the following: $L_{H1}$ with $<{PID}_{t},R_{t},h_{t}>$, $L_{K1}$ with $<{PID}_{t},R_{t},s_{t}>$ and $<{PID}_{t},{PK}_{t},x_{t}>$. Finally, β returns ${PK}_{t}$ to ${SA}_{1}$. Otherwise, β selects three random values $a_{t},b_{t},x_{t}\in Z_{n}^{*}$ and sets $R_{t}\leftarrow a_{t}P,h_{t}\leftarrow b_{t}$, $s_{t}\leftarrow ⊥,{PK}_{t}=x_{t}P+R_{t}$. β updates the following: $L_{H1}$ with $<{PID}_{t},R_{t},h_{t}>$, $L_{K1}$ with $<{PID}_{t},R_{t},⊥>$ and $<{PID}_{t},{PK}_{t},x_{t}>$. Finally, β returns ${PK}_{t}$ to ${SA}_{1}$.
- extract-partial-secret (${PID}_{t}$): Upon receiving an extract-partial-secret query for identity ${PID}_{t}$, if ${PID}_{t}={PID}^{*}$, β terminates the session. Otherwise, β checks $L_{H1}$ for an entry $<{PID}_{t},R_{t},s_{t}>$. If such a record exists, β returns $s_{t}$ to ${SA}_{1}$. Otherwise, β first performs a request-public-key (${PID}_{t}$) query and then returns $s_{t}$ to ${SA}_{1}$.
- extract-secret (${PID}_{t}$): Upon receiving an extract-secret query for identity ${PID}_{t}$, β searches $L_{K1}$ for $<{PID}_{t},{PK}_{t},x_{t}>$. If such a record exists, β returns $x_{t}$ to ${SA}_{1}$. Otherwise, β first performs an extract-partial-secret (${PID}_{t}$) query and then returns $x_{t}$ to ${SA}_{1}$.
- replace-public-key (${PID}_{t},{PK}_{t},{PK}_{t}^{\prime }$): Once β receives the query (${PID}_{t},{PK}_{t},{PK}_{t}^{\prime }$) from ${SA}_{1}$, it searches the list $L_{K1}$ for an entry $<{PID}_{t},{PK}_{t},x_{t}>$. If such a record exists, β sets ${PID}_{t}={PID}_{t}^{\prime }$ and $x_{t}=⊥$. Otherwise, β first performs a request-public-key (${PID}_{t}$) query and then sets ${PID}_{t}={PID}_{t}^{\prime }$ and $x_{t}=⊥$.
- super-sign (${PID}_{t},m_{t}$): Upon receiving a super-sign query with (${PID}_{t},m_{t}$) from ${SA}_{1}$, β searches for the following: $<{PID}_{t},R_{t},s_{t}>$ in the list $L_{H1}$ and $<{PID}_{t},{PK}_{t},x_{t}>$ in the list $L_{K1}$. Then, β generates a random value $c_{t}\in Z_{n}^{*}$ and computes the following: $V_{t}\leftarrow c_{t},T_{t}=V_{t}P-k_{t}({PK}_{t}+h_{t}{PK}_{KGC})$. Then, β returns the signature $\sigma_{t}=(U_{t},V_{t})$ to ${SA}_{1}$.

Finally, β outputs a forged but valid signature (${PID}_{t},m_{t},\sigma_{t}$). If ${PID}_{t}={PID}^{*}$, β terminates the simulation. Otherwise, β searches the tables $L_{H1}$ and $L_{K1}$ for the entries: $<h_{t},{PID}_{t},{PK}_{KGC}>,<k_{t},m,h_{t},{PK}_{t},T_{t}>,<{PID}_{t},s_{t},R_{t}>,<{PID}_{t},{PK}_{t},x_{t}>$. On the other hand, based on the Forking Lemma [27], through polynomial-time replay attacks under the same source of randomness but different hash oracle choices, it is possible to obtain two additional valid signatures. Eventually, three valid signatures are obtained: $\sigma_{t}^{\left(j\right)}=\left(U_{t}^{\left(j\right)},V_{t}^{\left(j\right)}\right),for\;j=1,2,3$. These signatures satisfy the following equation: $V_{t}^{\left(j\right)}=t_{t}^{\left(j\right)}+k_{t}^{\left(j\right)}\left({x_{t}+s}_{t}^{\left(j\right)}\right)=t_{t}^{\left(j\right)}+k_{t}^{\left(j\right)}\left(x_{t}+r_{t}+h_{t}^{\left(j\right)}s\right)\;mod\;n$, for j=1,2,3. Note that for ${SA}_{1}$ to win the first game, it must have never queried the extract-partial-secret or the super-sign oracle. Using the above three equations, β can solve for the three unknowns $x_{t},r_{t},s$ and s as the solution to a random instance of ECDLP, given by (P,Q=sP). So far, we have demonstrated that β can solve a given ECDLP instance. Next, we analyze the probability ${Succ}_{\beta }$ that β wins in Game 1.

- E1: β does not terminate in any extract-partial-secret queries.
- E2: ${SA}_{1}$ successfully forges a valid signature (${PID}_{t},m_{t},\sigma_{t}$).
- E3: The forged signature (${PID}_{t},\;m_{t},\sigma_{t}$) satisfies ${PID}_{t}={PID}^{*}$.

The corresponding probabilities of the three events mentioned above are given as follows: $Pr\left[E_{1}\right]\geq {\left(1-\frac{1}{q_{CU}+q_{H}}\right)}^{q_{EPS}},\mathrm{Pr}\left[E_{2}|E_{1}\right]\geq {Succ}_{{SA}_{1}},Pr\left[E_{3}|E_{1}\wedge E_{2}\right]\geq \frac{1}{q_{CU}+q_{H}}$, where $q_{CU},q_{H},{andq}_{EPS}$ represent the number of create-user queries, hash queries, and extract-partial-secret queries, respectively. In this case, the probability that β solves the given instance of ECDLP is as follows: ${Succ}_{\beta }=Pr\left[E_{1}\wedge E_{2}\wedge E_{3}\right]=Pr\left[E_{1}\right]Pr\left[E_{2}|E_{1}\right]Pr\left[E_{3}|E_{1}\wedge E_{2}\right]\geq \frac{1}{q_{CU}+q_{H}}{\left(1-\frac{1}{q_{CU}+q_{H}}\right)}^{q_{EPS}}$. Clearly, since ${Succ}_{{SA}_{1}}$ is non-negligible, β can solve ECDLP with a non-negligible probability ${Succ}_{\beta }$. This contradicts the assumed difficulty of ECDLP. □

**Theorem** **2.** *In the random oracle model, assuming that solving* ECDLP *is hard, the proposed certificateless signature scheme is existentially unforgeable against a Super Type-II adversary. That is, if there exists a Super Type-II adversary* ${SA}_{2}$*, which can query the random oracle model and win Game 2 with probability* ${Succ}_{{SA}_{2}}$*, then there exists an algorithm* β *that can solve a random instance of* ECDLP *in polynomial time with a success probability:* ${Succ}_{\beta }\;\geq \;\frac{1}{q_{CU}+{\;q}_{H}}{\left(1-\frac{1}{q_{CU}+q_{H}}\right)}^{q_{EPS}}{Succ}_{{SA}_{2}}$.

**Proof.**  Suppose there exists a Super Type-II adversary ${SA}_{2}$ that can break our certificateless signature scheme with a non-negligible probability ${Succ}_{{SA}_{2}}$. Then, we can construct a polynomial-time algorithm β that uses ${SA}_{2}$ to solve ECDLP. That is, β is given a random instance of ECDLP $\left(P,Q=x_{t}P\right)$. The goal of β  is to compute the secret key $x_{t}$. Initialization Phase: β selects an identity ${PID}^{*}$ as the challenge identity in Game 2. It sets the public key ${PK}_{KGC}$ and sends the master key s along with the parameters, $params=\left(G,P,{PK}_{KGC},H\right),$ to the adversary ${SA}_{2}$. β also maintains two lists, $L_{H2}$ and $L_{K2}$, to record oracle query responses. Next, during the query phase, β interacts with ${SA}_{2}$ and simulates the oracle model by responding to the adversary’s queries. Since the create-user, hash query, replace-public-key, and super-sign queries have already been defined in Theorem 1, we do not provide their descriptions here. In the following, we simulate the other oracle queries made by ${SA}_{2}$:

- request-public-key (${PID}_{t}$): When ${SA}_{2}$ queries the public key of identity ${PID}_{t}$, β proceeds as follows. If ${PID}_{t}\neq {PID}^{*}$, β generates two random values $r_{t},x_{t}\in Z_{n}^{*}$ and computes the following: $R_{t}=r_{t}P,\;h_{t}=H\left({PID}_{t},\;{PK}_{KGC}\right),s_{t}=r_{t}+h_{t}s\;mod\;n,\;{PK}_{t}=x_{t}P+R_{t}$. Then, β adds the tuples $<{PID}_{t},R_{t},h_{t}>,\;<{PID}_{t},s_{t},R_{t}>$ and $<{PID}_{t},{PK}_{t},x_{t}>$ to the lists $L_{H2}$ and $L_{K2}$. Finally, β returns ${PK}_{t}$ to ${SA}_{2}$. Otherwise, β selects a random value $r_{t}\in Z_{n}^{*}$ and sets $R_{t}=r_{t}P,h_{t}=H\left({PID}_{t},{PK}_{KGC}\right),s_{t}=r_{t}+h_{t}s\;mod\;n,{PK}_{t}=x_{t}P+R_{t}$. Then, β adds $<{PID}_{t},R_{t},h_{t}>$ to $L_{H2}$ and $<{PID}_{t},s_{t},R_{t}>,<{PID}_{t},{PK}_{t},⊥>$ to $L_{K2}$. Finally, β returns ${PK}_{t}$ to ${SA}_{2}$.
- extract-partial-secret (${PID}_{t}$): When ${SA}_{2}$ queries the partial private key for identity ${PID}_{t}$, β proceeds as follows: β searches for $<{PID}_{t},s_{t},R_{t}>$ in $L_{K2}$. If such a record exists, β returns $s_{t}$ to ${SA}_{2}$. Otherwise, β performs a request-public-key (${PID}_{t}$) query and returns $s_{t}$ to ${SA}_{2}$.
- extract-secret (${PID}_{t}$): When ${SA}_{2}$ queries the full private key of identity ${PID}_{t}$, β proceeds as follows: if ${PID}_{t}={PID}^{*}$, β terminates the session. Otherwise, β searches for $<{PID}_{t},{PK}_{t},x_{t}>$ in $L_{K2}$. If such a record exists, β returns $x_{t}$ to ${SA}_{2}$. Otherwise, β performs a request-public-key (${PID}_{t}$) query and then returns $x_{t}$ to ${SA}_{2}$.

Finally, ${SA}_{2}$ outputs a forged but valid signature (${PID}_{t},m_{t},\sigma_{t}$). If ${PID}_{t}={PID}^{*}$, the simulation stops. Otherwise, β searches in list $L_{K2}$ for $<{PID}_{t},s_{t},R_{t}>$ and $<{PID}_{t},{PK}_{t},x_{t}>$. Based on the Forking Lemma [27], if there is polynomial replay under the same randomness and different hash oracle model choices, ${SA}_{2}$ can generate another valid signature. Eventually, we obtain two valid signatures: $\sigma_{t}^{\left(j\right)}=(U_{t}^{\left(j\right)},V_{t}^{\left(j\right)})$ for j=1,2, satisfying the following equation: $V_{t}^{\left(j\right)}=t_{t}^{\left(j\right)}+k_{t}^{\left(j\right)}\left({x_{t}+s}_{t}^{\left(j\right)}\right)=t_{t}^{\left(j\right)}+k_{t}^{\left(j\right)}\left(x_{t}+r_{t}+h_{t}^{\left(j\right)}s\right)mod\;n$ for j=1,2. It is important to note that winning Game 2 requires that the oracle queries extract-secret and super-sign are never made. Using the two independent linear equations above, β can derive the two unknown values $r_{t}$ and $x_{t}$ and output them as the solution to the random ECDLP instance $\left(P,Q=x_{t}P\right)$. This, in turn, allows us to analyze the probability of β winning in Game 2. We introduce the events leading to success as follows:

- E1: β does not terminate in any extract-secret query.
- E2: ${SA}_{2}$ successfully forges a valid signature (${PID}_{t},m_{t},\sigma_{t}$).
- E3: The forged signature (${PID}_{t},m_{t},\sigma_{t}$) satisfies ${PID}_{t}={PID}^{*}$.

The probabilities of these events are given as follows: $Pr\left[E_{1}\right]\geq {\left(1-\frac{1}{q_{CU}+q_{H}}\right)}^{q_{ES}},\mathrm{Pr}\left[E_{2}|E_{1}\right]\geq {Succ}_{{SA}_{1}},Pr\left[E_{3}|E_{1}\wedge E_{2}\right]\geq \frac{1}{q_{CU}+q_{H}}$, where $q_{CU},\;q_{H},\;and\;q_{ES}$ denote the number of create-user queries, hash queries, and extract-secret queries, respectively. Thus, the probability that β solves the given instance of ECDLP is as follows: ${Succ}_{\beta }=Pr\left[E_{1}\wedge E_{2}\wedge E_{3}\right]=Pr\left[E_{1}\right]Pr\left[E_{2}|E_{1}\right]Pr\left[E_{3}|E_{1}\wedge E_{2}\right]\geq \frac{1}{q_{CU}+q_{H}}{\left(1-\frac{1}{q_{CU}+q_{H}}\right)}^{q_{ES}}$. Since ${Succ}_{{SA}_{2}}$ is non-negligible, β is able to solve ECDLP with non-negligible probability, ${Succ}_{\beta }$, contradicting the difficulty of solving ECDLP. □

In summary, the proposed ECAE scheme achieves existential unforgeability under the random oracle model based on the difficulty assumption of the Elliptic Curve Discrete Logarithm Problem (ECDLP). Through the proof of formal security, it effectively resists two types of adversaries and defends against advanced attacks such as public key replacement and key escrow, thereby providing provable security guarantees for NDN-IoT environments.

### Informal Security Analysis

Anonymity: During communication, the true identity of the terminal device is hidden behind a pseudonym, such as ${PID}_{i}=\{{MID}_{i},T_{i}\}$, where ${MID}_{i}=H1\left(T_{i},s{AID}_{i}\right)⊕{ID}_{i},$ and ${AID}_{i}={ID}_{i}⊕N_{i}$. Here, $T_{i}$ represents the validity period of the pseudonym. Each terminal device uses a pseudonym ${PID}_{i}$ generated by the KGC for communication. Apart from the KGC, no adversary can retrieve the real identity of the terminal device from the pseudonym.

Unlinkability: During communication, the terminal device uses different pseudonyms for each interaction. Each message is signed using a different pseudonym and its corresponding private key. There is no connection between the new and old pseudonyms. Therefore, an attacker cannot infer the terminal device from the new pseudonym, ensuring the unlinkability of the scheme.

Message Authentication: In our scheme, every message generated by a terminal device must be signed before being sent to a nearby NDN router. The NDN router can verify the signature to ensure that the message has not been tampered with or forged by an attacker or an unauthorized device. Additionally, the ECAE scheme provides existential unforgeability against both Type-I and Type-II adversaries. Therefore, the proposed scheme meets the security requirements for message authentication.

Conditional Traceability: When a terminal device malfunctions or causes an incident, the KGC can trace the real identity of the user. The KGC retrieves $\left\{{PID}_{i},M_{i},{AID}_{i}\right\}$ from its secure database and computes ${ID}_{i}={AID}_{i}⊕sM_{i}$ to obtain the real identity of the malicious user. Since s is the master private key of the KGC, only the KGC has access to this information. Thus, only the KGC can trace the real identity of the terminal device, while no other entity can do so.

Non-repudiation: Based on the analysis above, if a terminal device denies certain information, the KGC can identify its real identity through the pseudonym of the malicious device. Therefore, no device can deny its signature on a message.

Resistance to Replay Attacks: A replay attack occurs when an attacker records or reuses communication messages to deceive the system. In the proposed scheme, the interactions between the ED, NDN router, and KGC incorporate random numbers and timestamps. The recipient can detect whether a message is a replayed one and effectively filter out any illegitimate replayed messages.

Perfect Forward Secrecy: Suppose an attacker obtains the private key ${SK}_{i}={\;d}_{i}+h_{2i}x_{i}$ of a terminal device, ${ED}_{i}$. Since the temporary random number $u_{i}\in Z_{q}^{*}$ used by ${ED}_{i}$ during each signing session is independent of the private key and the signature $\sigma_{i}=(U_{i},V_{i})$ includes $V_{i}=u_{i}+h_{3i}{SK}_{i}$, which depends on $u_{i}$, the attacker cannot derive $u_{i}$ from any historical session, even if ${SK}_{i}$ is exposed. This is because recovering $u_{i}$ from $U_{i}=u_{i}P$ requires solving the *ECDLP*. Similarly, in the aggregate signature, σ=(U, V), where $V=\sum_{i=1}^{n}V_{i}$, the value also depends on the temporary random numbers used in each session. This further ensures that even if a terminal device’s key is compromised, the confidentiality of previous sessions remains intact. Therefore, the ECAE scheme possesses the property of perfect forward secrecy.

## 6. Performance Analysis

In this section, the ECAE scheme will be compared with the schemes proposed by Huang et al. [11], Yang et al. [15], Karati et al. [16], Thumbur et al. [17], and Zhu et al. [23] in terms of security features, the number of cryptographic operations, computational delay, and communication overhead. Due to its high efficiency and robust security properties, the Curve25519 elliptic curve has been widely adopted in various fields [28,29]. Therefore, this experiment selected Curve25519 as the underlying elliptic curve, with a security parameter of λ = 256. The curve is defined over the prime field $\;p=2^{255}-19$, and its equation is given by $E:y^{2}\;=\;x^{3}\;+\;{48662x}^{2}\;+\;x$, where the x-coordinate of the base point P is 9.

### 6.1. Security Functionality Comparison

Table 2 presents a functional comparison between the ECAE scheme and other existing schemes. Notably, many current signature schemes do not ensure the anonymity of message sources, which may allow attackers to easily trace the origin of messages, thereby compromising user privacy. In addition, the group signature scheme proposed by Huang et al. [11] in recent years has improved authentication efficiency. However, its resistance to man-in-the-middle attacks is inadequate, giving attackers opportunities to insert themselves between communicating parties, enabling eavesdropping, message tampering, or impersonation. By comparison, the scheme proposed by Karati et al. [16] appears relatively outdated. It lacks non-repudiation and unlinkability, making it vulnerable to signature forgery attacks, which compromise the integrity and authenticity of information. The schemes proposed by Karati et al. [16], Thumbur et al. [17], and Zhu et al. [23] share several common shortcomings, such as the absence of a timely signature update mechanism, making them ineffective against replay attacks. Bisheh-Niasar et al. [19] adopted the Edwards curve signature algorithm and focused primarily on hardware architecture. However, their scheme remains vulnerable to man-in-the-middle and replay attacks. Unlike these schemes, the solution proposed in this paper comprehensively meets all the security requirements listed in the table, offering a more complete and efficient approach to overcoming the limitations of existing schemes.

### 6.2. Computation Delay

We simulated these methods on two Ubuntu 16.04 devices using the OpenSSL library [30], GMP library [31], and PBC library [32]. The terminal device hardware used was a RASPBERRY PI 3B+ with 1 GB of LPDDR2 SDRAM and a 1.4 GHz BCM2837B0 system-on-chip. The AG hardware used a computer with 4 GB of RAM and an INTEL(R) CELERON (R) J1900 CPU.

In the six schemes compared, the cryptographic operations used included hash operations, point multiplication, point addition, XOR operations, exponentiation, and bilinear pairing operations, which are denoted as follows: Has, Mul, Add, Xor, EXP, and BPA. The execution time for each cryptographic operation is shown in Table 3.

**Figure 5:**
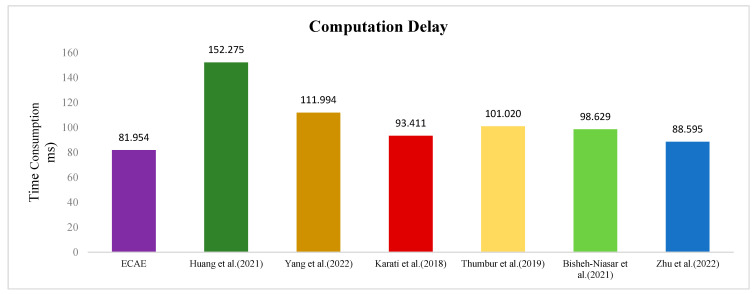
Comparison of computation delay [11,15,16,17,19,23].

Table 4 provides a comparison of the number of cryptographic operations and their execution times across different schemes. It can be observed that the ECAE scheme exhibits the shortest overall execution time on the ED. Figure 5 shows a comparison of computational delays between the schemes. The total computation time of the ECAE scheme is 46.18% lower than Huang et al.’s [11] scheme, 26.82% lower than Yang et al.’s scheme [15], 12.27% lower than Karati et al.’s scheme [16], 19.07% lower than Thumbur et al.’s scheme [17], 20.35% lower than Bisheh-Niasar et al.’s scheme [19], and 7.50% lower than Zhu et al.’s scheme [23].

In Section 4, a comprehensive security analysis of the ECAE scheme is provided to validate its resistance against typical attacks. Through this security analysis, we define the attacks we can defend against as known attacks, while other potential attacks, due to their unpredictability, are categorized as unknown attacks. It is assumed that unknown attacks may interfere with the authentication process of these seven schemes, whereas known attacks do not. In the case of known attacks, each scheme exhibits fixed computational delays; however, in the case of unknown attacks, the computational delay may vary. To assess the impact of this uncertainty on performance, simulations were conducted using C++ experiments, evaluating the performance of the authentication schemes under unknown attack conditions. By adjusting the ratio of unknown attacks to known attacks, the computational delay for each scheme can be measured. The performance measurement parameter is defined as the average successful computational delay calculated by Equation (4). Additionally, delay_known_ represents the computational delay under known attacks, while delay_unknown_ represents the computational delay under unknown attacks, which is a random value.

(4) $$\begin{array}{c} {delay}_{average}=\frac{{delay}_{unknown}\;*\;{times}_{unknown}+{delay}_{unknown}\;*\;{times}_{unknown}}{{times}_{known}}\;\\ where\;{times}_{unknown}={ratio}_{unknown}\;*\;{times}_{all-attack} \end{array}$$

**Figure 6:**
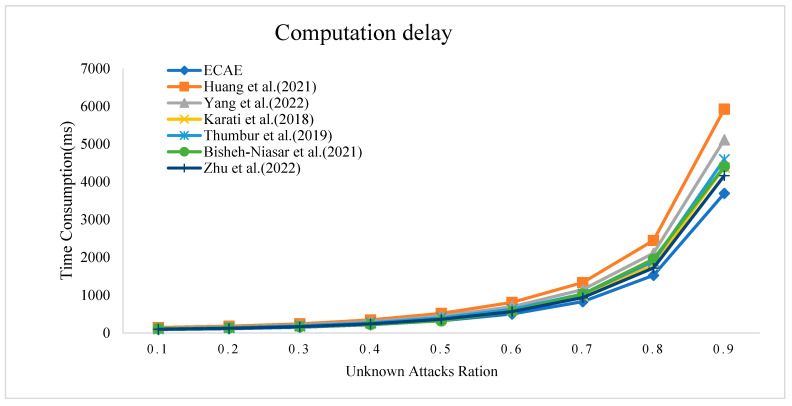
Computation delay under a ration of unknown attacks [11,15,16,17,19,23].

Figure 6 shows the relationship between the average computational delay of each scheme and the proportion of unknown attacks. The vertical axis represents the average computational delay, and the horizontal axis represents the proportion of unknown attacks out of the total attacks. The seven schemes are represented by curves of different colors and shapes. As seen in Figure 6, with the increase in the proportion of unknown attacks, the average computational delay of each scheme gradually increases. Notably, the ECAE scheme exhibits the smallest computational delay as the proportion of unknown attacks increases. Through a comprehensive analysis of efficiency and security, it was concluded that the proposed scheme has significant advantages in both aspects.

### 6.3. Communication Overhead

**Figure 7:**
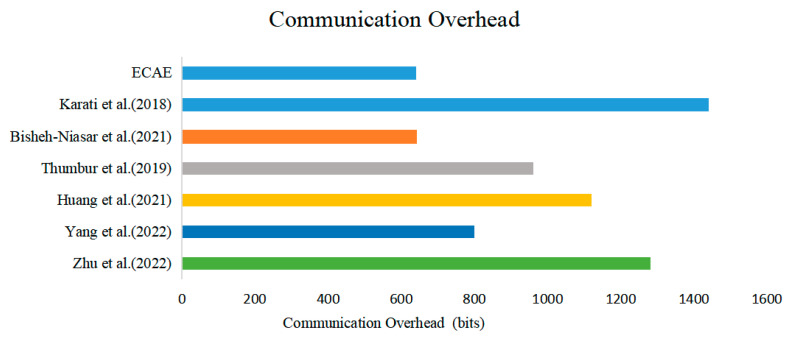
Comparison of communication overheads across different schemes [11,15,16,17,19,23].

In the NDN packet forwarding mechanism, the transmission of an Interest packet triggers the entire data transfer process. The Interest packet propagates through the network, and each router makes intelligent forwarding decisions based on the data name in the Interest packet. Once the Interest packet reaches the producer, the data packet is generated and accompanied by a digital signature to ensure data integrity and source authenticity. These data packets are transmitted along the reverse path of the Interest packet and are forwarded by routers until they reach the initiator of the Interest. Therefore, the length of the signature is an important factor in determining the communication costs. In this experiment, it was assumed that the communication usage for point multiplication in $S_{M}$ was 320 bits, and the communication usage for the numbers in $S_{s}$ was 160 bits. Table 5 presents the analysis of the signature length in the seven schemes. Figure 7 shows a comparison of the total communication overheads across the seven schemes.

By analyzing Table 5 and Figure 7, it can be concluded that the signature lengths in both the ECAE scheme and the scheme proposed by Bisheh-Niasar et al. are the shortest. This is because both schemes employ fewer point multiplication operations during the signature generation process and emphasize the inclusion of minimal data in the signature design. Therefore, from the perspective of memory and communication overheads, the ECAE scheme demonstrates a notable performance advantage.

## 7. Conclusions and Future Work

The research results show that the signature algorithms used in NDN to ensure packet security still have limitations in terms of security and compatibility with IoT devices. To effectively address these issues, this paper proposes an efficient certificateless aggregate signature scheme in the NDN-IoT environment. By utilizing low-cost elliptic curve cryptosystems and conventional hash functions, the signature verification process in the NDN architecture is optimized, thereby reducing the signature verification time overhead and the cache overhead of NDN routers, easing the burden of certificate management and achieving high compatibility with resource-constrained IoT devices. Experimental data show that the ECAE scheme reduces the total computation time by up to 46.18% compared to existing schemes. Furthermore, even in unknown attack scenarios, the ECAE scheme maintains stable performance, with a signature communication usage of only 640 bits, significantly reducing system resource requirements. To highlight the advantages of the ECAE scheme, a comprehensive security evaluation was also conducted. The security of this scheme is based on the ECDLP and has been verified under the random oracle model, proving that it can effectively resist Type I/II attacks while also providing multiple security properties such as anonymity and unforgeability. The simulation results show that, with relatively low computational delay and communication overhead, the ECAE scheme offers all the security functions provided by other schemes.

Our paper is based on the random oracle model (ROM). Although the ROM offers a concise and powerful framework for the security analysis of the ECAE scheme, it relies on several idealized assumptions that may not fully reflect real-world security scenarios. First, the ROM treats hash functions as perfect random functions, while real-world hash algorithms (e.g., SHA-3) are deterministic and may exhibit structural properties that attackers could exploit in targeted attacks, such as length extension or algebraic manipulation. Second, proof of security in the ROM does not directly provide concrete security guarantees in the standard model, especially in scenarios where hash functions are maliciously constructed or are subject to side-channel leakage. For example, if an adversary can extract the internal state of a hash function via physical side channels, the “unpredictability” assumption inherent to the ROM is broken. Moreover, transitioning from the ROM to the standard model typically necessitates stronger computational assumptions (e.g., DDH or LWE) or more complex cryptographic constructions, which may lead to significant performance degradation (e.g., increased group operations or larger key sizes). Although the ECAE scheme has been proven to be secure under the ROM, future work should explore the selection of hash functions that align with the abstract properties required by the ROM, develop provably secure alternatives in the standard model, and address potential threats such as the impact of quantum computing on the security of hash functions.

Furthermore, ECAE’s design—featuring constant-size aggregated signatures, pairing-free verification, and lightweight ECC operations—naturally supports high scalability under extreme network loads and massive packet volumes since routers can arbitrarily verify many signatures in constant time and end devices incur only minimal per-message overheads. However, actual performance under bursts of heterogeneous traffic still depends on factors such as aggregation window size, hardware parallelism, network topology, and the distribution of the Key Generation Center (KGC). Therefore, our future work will include large-scale simulations and testbed deployments to characterize latency–throughput trade-offs across diverse topologies, optimize dynamic aggregation intervals and parallel verification strategies, and explore distributed KGC architectures with adaptive parameter tuning so as to maintain millisecond-level verification latency even as the numbers of producers and data packets scale into the tens or hundreds of thousands.

In future research, we plan to explore the synergy of this scheme with other security mechanisms to build a more comprehensive NDN-IoT security architecture to meet the growing network threats. Additionally, we will investigate its practical performance under real-world hash functions and address performance bottlenecks in high-traffic NDN-IoT environments through systematic solutions. Beyond this, we aim to design extended functionalities such as group signatures and ring signatures to enhance adaptability in diverse application scenarios.

## Figures and Tables

**Table 1:** Commonly used identifiers.

Parameter	Meaning
KGC	Key Generation Center
NDN Router	NDN Router
ED	End Device
${ED}_{i}$	The ith End Device
${ID}_{i}$	The Real Identity of the ith End Device
${PID}_{i}$	The Pseudonym of the ith End Device
s	Private Key of KGC
$P_{pub}$	Public Key of KGC
${SK}_{i}$	Private Key of the ith End Device
${PK}_{i}$	Public Key of the ith End Device
$t_{i}$	Current Timestamp
$T_{i}$	Validity Period of the Pseudonym
σ	Single Signature
$\sigma_{i}$	Aggregate Signature
⊕	XOR Operator

**Table 2:** Comparison of security functionality.

	Anonymity	Non-Repudiation	Unlinkability	Signature Forgery Attack	MITM Attack	Replay Attack
Huang et al. [11]	×	√	√	√	×	√
Yang et al. [15]	×	√	√	√	√	√
Karati et al. [16]	×	×	×	×	√	×
Thumbur et al. [17]	×	√	√	√	√	×
Bisheh-Niasar et al. [19]	×	√	√	√	×	×
Zhu et al. [23]	×	√	√	√	√	×
ECAE (Ours)	√	√	√	√	√	√

**Table 3:** Execution time of basic cryptographic operations (ms).

Operation	Description	BCM2837B0	Intel J1900
$T_{\mathrm{Has}}$	Execution time of the hash function	0.0729 ms	0.0023 ms
$T_{\mathrm{Mul}}$	Execution time of ECC point multiplication	23.4405 ms	2.2260 ms
$T_{\mathrm{Add}}$	Execution time of ECC point addition	0.1652 ms	0.0288 ms
$T_{Xor}$	Execution time of XOR operation	0.1650 ms	0.0490 ms
$T_{EXP}$	Execution time of exponentiation	3.3280 ms	0.0390 ms
$T_{BPA}$	Execution time of bilinear pairing	48.6600 ms	5.2750 ms

**Table 4:** Comparison of the number of cryptography operations and execution time.

Scheme	Executor	Has	Mul	Add	Xor	EXP	BPA	Total
Huang et al. [11]	User	1	4	2	0	7	0	152.2749 ms
	Server	0	6	7	0	4	4	
Yang et al. [15]	User	3	4	1	1	1	0	111.9935 ms
	Server	3	4	2	1	0	1	
Karati et al. [16]	User	1	3	0	0	3	0	93.4114 ms
	Server	1	1	0	0	6	2	
Thumbur et al. [17]	User	2	4	2	0	0	0	101.0198 ms
	Server	2	3	2	0	0	0	
Bisheh-Niasar et al. [19]	User	3	4	1	0	0	0	98.6290 ms
	Server	1	2	1	0	0	0	
Zhu et al. [23]	User	4	3	4	0	0	0	88.5952 ms
	Server	3	3	3	0	0	2	
ECAE	User	1	3	1	0	0	0	81.9544 ms
	Server	4	5	6	0	0	0	

**Table 5:** Signature length analysis.

Scheme	Signature Length
Huang et al. [11]	$3S_{M}+S_{s}$
Yang et al. [15]	2$S_{M}+S_{s}$
Karati et al. [16]	$4S_{M}+S_{s}$
Thumbur et al. [17]	$3S_{M}$
Bisheh-Niasar et al. [19]	2$S_{M}$
Zhu et al. [23]	$3S_{M}+{\;2S}_{s}$
ECAE	$2S_{M}$

## Data Availability

The data are contained within the article.
